# Assessment of the effect of the corticotomy-assisted orthodontic treatment on the maxillary periodontal tissue in patients with malocclusions with transverse maxillary deficiency: a case series

**DOI:** 10.1186/s12903-018-0625-0

**Published:** 2018-10-03

**Authors:** Magdalena Sulewska, Ewa Duraj, Beata Bugała-Musiatowicz, Emilia Waszkiewicz-Sewastianik, Robert Milewski, Jan K. Pietruski, Eugeniusz Sajewicz, Małgorzata Pietruska

**Affiliations:** 1 Department of Periodontal and Oral Mucosa Diseases, Medical University of Białystok, ul. Waszyngtona 13, 15-269 Białystok, Poland; 2 Dental Practice, ul. Żeromskiego 1A/1U, 15-349 Białystok, Poland; 3 Dental Practice, ul. Waszyngtona 1/34, 15-269 Białystok, Poland; 4 Department of Statistics and Medical Informatics, Medical University of Białystok, ul. Szpitalna 37, 15-295 Białystok, Poland; 5 Department of Biocybernetics and Biomedical Ingeenering, Białystok University of Technology, ul. Wiejska 45c, 15-351 Białystok, Poland

**Keywords:** Corticotomy, Orthodontics, Periodontics, Malocclusion

## Abstract

**Background:**

The aim of the study was to assess the effect of corticotomy–assisted orthodontic treatment on soft tissue clinical parameters in patients with malocclusions with transverse maxillary deficiency.

**Methods:**

The study included 20 generally healthy adult individuals with malocclusion, who underwent a corticotomy-assisted orthodontic treatment in maxilla. During the corticotomy performed after full-thickness flap elevation, only the buccal cortical plate was cut with the use of OTS-7, OTS7–4, OTS7-3 ultrasound tips of the piezosurgery device (Mectron s. p. a., Italy). A clinical examination was performed prior to the corticotomy procedure, then repeated – 3, 6, 9 and 12 months after the procedure. The following parameters were assessed: FMPI (full mouth plaque index), FMBOP (full mouth bleading on probing), PD (probing depth), CAL (clinical attachment level), GR (gingival recession height), RW (recession width), PH (papilla height), PW (papilla width), BS (bone sounding), biotype and KT.

**Results:**

There was a statistically significant reduction in PD (mean difference: 0.06; 95% Cl: − 0.33, − 0.18), CAL (mean difference: 0.07; 95% Cl: − 0.33, − 0.19), PH (mean difference: 0.26; 95% Cl: − 0.47, 0.05) and BS (mean difference: 0.13; 95% Cl: − 0.41, − 0.14) after the treatment. Statistically significant changes were also noted in relation to KT (mean difference: 0.17; 95% Cl: − 0.07, 0.27) and biotype (mean difference: 0.07; 95% Cl: 0.26, 0.39), which thickness increased significantly after the treatment. No statistically significant differences were observed in GR, RW and PW.

**Conclusions:**

The corticotomy–assisted orthodontic treatment did not jeopardize the periodontal clinical status in maxilla. There is a need for further studies on a larger number of patient to compare the clinical findings with a control group as well as in patients with conventional orthodontic treatment in a longer follow-up time to find out more about the post-treatment periodontal tissue changes and stability.

## Background

The introduction of corticotomy-assisted orthodontics provided new solutions to some limitations in orthodontic treatment [1]. Corticotomy-assisted orthodontics induces a state of increased tissue turnover and transient osteopenia, followed by a faster rate of orthodontic tooth movement [2]. The corticotomy technique has several advantages, including faster tooth movement, shorter treatment time, safer expansion of constricted arches, enhanced post-orthodontic treatment stability, and an extended envelope of tooth movement [2–4].

The accelerated tooth movement technique was described for the first time by Köle [5]. The method involved the formation of bone blocks by means of vertical inter-root corticotomy from the vestibular and lingual side as well as supra-apical osteotomy, which allowed for quicker movement of bony blocks along with the teeth without any potential adverse consequences for the periodontium. In 1990, Gantes et al. [6] used Köle’s modified technique, in which osteotomy was replaced by horizontal corticotomy, and concluded that the corticotomy procedure caused minimal changes in the periodontal attachment apparatus and allowed for a reduction in treatment duration of up to 50%. Wilcko et al. [7] described the periodontally accelerated osteogenic orthodontics (PAOO) technique. This new surgical technique included buccal and lingual full-thickness flaps, selective partial decortication of the cortical plates, and concomitant bone grafting. In a subsequent study Wilcko and co-workers found that orthodontic movement is not a simple repositioning of single tooth-bone units, but is a cascade of physiological events leading to bone healing [8–10]. This process, called by Frost [11] the regional accelerated phenomenon (RAP), and described in the periodontal literature by Yaffe et al. [12], assumes that healing is a complex physiologic process with dominating features involving accelerated bone turnover and decreases in regional bone densities. RAP is not a separate healing event, but it can expedite hard and soft tissue healing stages two- to tenfold [2, 13]. After corticotomy, demineralization occurs in the alveolar bone and the remaining collagenous matrix of bone is transported with the tooth during its movement [2]. The matrix then remineralizes after the orthodontic movement [7, 8]. Computerized tomography imaging, animal studies, and histological evaluation support the hypothesis of reversible osteopenia that is responsible for rapid tooth movement in corticotomy-assisted orthodontics [9, 14, 15]. In 2007, Vercellotti and Podesta [16] reported a microsurgical technique in which cuts are made around each tooth root with only one full thickness flap on the side corresponding to the direction of dental movement. In this monocortical tooth dislocation and ligament distraction technique (MTDLD) dental movement occurs via dislocation of the root and cortical bone together, without periodontal ligament compression and bone resorption. Then in 2009 Dibart et al. [17] proposed, minimally invasive technique combining microincisions with selective tunneling and piezoelectric incisions between roots with consecutive hard- or soft-tissues grafting.

The literature gathers the evidence of successful corticotomy as an aid to orthodontic treatment and doesn’t report it’s negative effects, however, there is lack of a detailed analysis of the changes that occur in the periodontal tissues during and after treatment [2, 7–9, 18–22]. That is why the aim of this study was to assess the effects of the corticotomy-assisted orthodontic treatment on clinical status of soft tissues in patients with malocclusion.

## Methods

The study included 20 generally healthy adult individuals (10 female and 10 male) aged 19 to 35 with Class I and II malocclusion which a common feature was transverse maxillary deficiency.

A full aesthetic, functional and orthodontic analysis was done prior to the treatment. A periodontal examination was conducted along with photographic and radiographic documentation including orthopanthomogram, cephalometric x-ray as well as cone beam computed tomography. The patients were told about the advantages, disadvantages and risk involved in the corticotomy-assisted orthodontic treatment. All the patients gave their written informed consent for treatment and participation in the study. The study was carried out in accordance with the Helsinki Declaration of 1975, as revised in 2000, and was reviewed and approved by the local ethical committee (Ethics Committee Nr.: R-I-002/344/2011).

### Inclusion criteria

Voluntary participation; Legal adult (> 18 years old); Non-smoking; Generally healthy; Malocclusion with transverse maxillary deficiency; Indications for upper arch expansion during treatment; Good oral hygiene and motivation at screening quantified as: FMPI (full mouth plaque index) < 20%, FMBOP (full mouth bleading on probing) < 20%.

### Exclusion criteria

Periodontal disease; Oral mucosa lesions; Bisphosphonate and long-term corticosteroid therapy; Current therapy with: anti-epileptic drugs, contraceptives, estrogen, antihistamine drugs, calcitonin, vitamin D; Alcohol and/or drug addiction; Presence of periapical endo-perio lesions; Severe gingival recession; Pregnancy, breast feeding; Previous orthodontic treatment; Previous root resorption; Inability to commit to one-year follow-up.

#### Surgical procedure

One day prior to the surgery thin arch self-ligating brackets (System Damon, Ormco, Orange, CA, USA) were bonded without placing the archwire. Amoxicillin at a dose of 1 g and ibuprofen at a dose of 200 mg were administered before the surgical procedure. The surgery was done in maxilla under local anesthesia with 4% articaine (Ubistesin forte, 3 M ESPE, USA). The mucoperiosteal flap was elevated up to the point above the apical parts of roots following modified papilla preservation technique as well as performing vertical releasing incisions [23]. Then osteotomy of the buccal cortical plate of the alveolar process was performed by using OTS7, OTS7–4, OTS7-3 ultrasound tips of the piezosurgery device (Mectron s. p. a., Italy). The extension of the osteotomy was determined by the mesio-distal dimension of the teeth roots as well as by the position of the apexes of roots. In order to avoid interproximal bone picks resorption, the vertical cuts ended 5 mm apically from the crest and then Y-shape spread towards the neighboring teeth. The horizontal corticotomy was performed approximately 2–4 mm apically above the root apexes. The depth of the cuts was limited to the thickness of the cortical plate. The repositioned flap was sutured with non-resorbable monofilament 5.0 and 6.0 sutures (Resolon, Resorba Medical GmbH, Germany). Amoxicillin 1 g 2×/day for 7 days, ibuprofen 200 mg 3×/day, mouth rinsing with chlorhexidine (0.10% Eludril, Pierre Fabre Sante, France) 2×/day were prescribed and gentle tooth brushing in the surgical area for two weeks was recommended to the patients. The supragingival plaque was cleaned out 7 and 14 days after the surgery. The sutures were removed 14 days post-op.

#### Orthodontic treatment

Subsequently after the corticotomy, initial orthodontic wires (0.012 or 0.014 Cooper Ni-Ti) were placed (Ormco, Orange, CA, USA). The follow-ups were performed every 2 weeks for the first three months of treatment, then every 4–6 weeks. The arches were fully leveled and aligned by using increasing sizes of nickel-titanium alloy archwires. The subsequent stages of treatment involved the use of: 0.018 Cooper Ni-Ti wires, replaced with rectangular ones. The therapy was completed with 0.019 × 0.025 steel archwires. The total time of treatment in both jaws took 9 to 12 months. Once the treatment was completed, a permanent retainer was bonded to the lower incisors and canines, while a removable retainer was provided for the upper arch.

#### Clinical examination

The clinical examination was performed in maxilla prior to the treatment, then 3, 6, 9 and 12 months after the surgery in accordance with the established protocol. The measurements were done using a manual PCP UNC 15 periodontal probe (Hu-Friedy, Chicago, IL, USA) by one calibrated investigator. The total number of examined teeth was 159. The clinical status of the surgical sites was photographically documented during the subsequent appointments.

The following clinical parameters were evaluated: FMPI (full mouth plaque index), FMBOP (full mouth bleading on probing), PD (probing depth), CAL (clinical attachment level), GR (gingival recession height), RW (recession width), PH (papilla height), PW (papilla width), BS (bone sounding), biotype and KT (keratinized tissue).

Clinical parameters were assesed as follows:

- PD (probing depth) and CAL (clinical attachment level) - at six points for each tooth,
- GR (gingival recession, height) - measured at mid-buccal aspect of the tooth from the CEJ to the most apical extension of gingival margin,
- RW (recession width) – mesio-distal dimention of denudated root surface measured at CEJ level,
- PH (papilla height) - measured on the midline of papilla from PW level to the tip of papilla,
- PW (papilla width) - measured at the level of CEJ of adjacent teeth,
- BS (bone sounding) - distance from the gingival margin to the the alveolar crest, measured using a periodontal probe under anesthesia, on the interproximal surfaces of teeth,
- biotype - gingival thickness - measured under anesthesia at mid-facial aspect of the tooth on a long axis 1 mm apicaly fom the bottom of the sulcus with the use of K-file 25 ISO with a silicone marker,
- KT (keratinized tissue) - measured from the most apical point of gingival margin to the mucogingival junction.

All measurements were rounded to the nearest 0.5 mm.

#### Statistical analysis

All continous variables were tested for normal distribtion by the Kolmogorov–Smirnov test, with Lilliefors corretion and Shapiro-Wilk test. Normal distribution of the quantitative variables was not found. The Friedman ANOVA non-parametric test was used for multiple comparisons to compare more than two related variables. 95% coinfidence intervals were also calculated for differences between baseline and 12 months post-op. Statistical significance was determined at *p* < 0.05. All calculations were performed using Statistica 10.0 software (StatSoft, USA).

## Results

FMPI and FMBOP remained at similar levels throughout the treatment with a tendency to decrease during retention (Table 1). There was a statistically significant reduction in mean PD and CAL after corticotomy-assisted orthodontic treatment as compared with the baseline. PD values decreased from 2.74 ± 0.57 mm to 2.48 ± 0.51 mm and CAL values decreased from 2.75 ± 0.57 mm to 2.49 ± 0.51 mm respectively. Mean pre- and post-treatment PD and CAL values are shown in Table 2.

**Table 1:** Full mouth plaque index (FMPI) and full mouth bleeding on probing (FMBOP) before and after orthodontic treatment

Parameter	[%]	Time of observation [months]		Difference between baseline and 12 months post-op	*p*-value (Friedman ANOVA)	Mean diff. (95% Cl) between baseline and 12 months post-op
FMPI	x ± SD	Baseline	17.33 ± 2.11	−0.35%	*p* = 0.28	0.86 (−1.29, 0.43)
		3	19.28 ± 2.52			
		6	20.13 ± 2.11			
		9	19.37 ± 2.17			
		12	16.90 ± 2.25			
FMBOP	x ± SD	Baseline	13.44 ± 1.87	−0.20%	*p* = 0.30	0.43 (−0.63, 0.23)
		3	15.07 ± 1.78			
		6	17.15 ± 1.80			
		9	15.79 ± 1.75			
		12	13.24 ± 1.75			

*x ± SD* mean values and standard deviation

*Mean diff.* mean difference

*Cl* Confidence interval

**Table 2:** Probing depth (PD), clinical attachment level (CAL), bone sounding (BS) before and during subsequent follow-up assessments

Parameter	[mm]	Time of observation [months]		Difference between baseline and 12 months post-op	*p*-value (Friedman ANOVA)	Mean diff. (95% Cl) between baseline and 12 months post-op
PD	x ± SD	Baseline	2.74 ± 0.57	− 0.26 mm	*p* < 0.001	0.07 (− 0.33, − 0.18)
		3	2.54 ± 0.64			
		6	2.58 ± 0.55			
		9	2.62 ± 0.57			
		12	2.48 ± 0.51			
CAL	x ± SD	Baseline	2.75 ± 0.57	−0.26 mm	*p* < 0.001	0.07 (− 0.33, − 0.19)
		3	2.55 ± 0.64			
		6	2.59 ± 0.55			
		9	2.61 ± 0.58			
		12	2.49 ± 0.51			
BS	x ± SD	Baseline	4.76 ± 0.94	−0.27 mm	*p* < 0.001	0.13 (−0.41, − 0.14)
		3	4.65 ± 0.84			
		6	4.82 ± 0.82			
		9	4.65 ± 0.88			
		12	4.49 ± 0.77			

*x ± SD* mean values and standard deviation

*Mean diff.* mean difference

*Cl* Coinfidence interval

There was also a statistically significant reduction in papilla height and bone sounding after the treatment. Reduced papilla height (PH) was reflected in the bone sounding (BS) value, which decreased by 0.27 mm post-treatment (Tables 2 and 3).

**Table 3:** Biotype, papilla width (PW), papilla height (PH), gingival recession (GR), recession width (RW) and keratinized tissue (KT) before and after orthodontic treatment

Parameter	[mm]	Time of observation [months]		Difference between baseline and 12 months post-op	*p*-value (Friedman ANOVA)	Mean diff. (95% Cl) between baseline and 12 months post-op
Biotype	x ± SD	Baseline	1.71 ± 0.52	+ 0.32 mm	*p* < 0.0001	0.07 (0.26, 0.39)
		3	1.95 ± 0.54			
		6	1.89 ± 0.56			
		9	1.94 ± 0.59			
		12	2.03 ± 0.47			
PW	x ± SD	Baseline	3.75 ± 0.92	−0.21 mm	NS	0.29 (−1.11, −0.53)
		3	3.60 ± 1.17			
		6	3.77 ± 0.95			
		9	3.80 ± 1.13			
		12	3.54 ± 1.50			
PH	x ± SD	Baseline	4.82 ± 1.16	−0.82 mm	*p* < 0.0001	0.26 (− 0.47, 0.05)
		3	4.16 ± 1.23			
		6	4.52 ± 0.85			
		9	4.51 ± 1.14			
		12	4.00 ± 1.60			
GR	x ± SD	Baseline	0.13 ± 0.47	−0.06 mm	NS	0.05 (−0.11, −0.01)
		3	0.09 ± 0.39			
		6	0.08 ± 0.34			
		9	0.09 ± 0.36			
		12	0.07 ± 0.32			
RW	x ± SD	Baseline	0.21 ± 0.75	−0.11 mm	NS	0.08 (−0.20, −0.03)
		3	0.17 ± 0.65			
		6	0.16 ± 0.66			
		9	0.14 ± 0.61			
		12	0.10 ± 0.49			
KT	x ± SD	Baseline	5.02 ± 1.79	−0.10 mm	*p* = 0.0039	0.17 (−0.07, 0.27)
		3	5.21 ± 1.65			
		6	5.08 ± 1.75			
		9	5.12 ± 1.83			
		12	5.12 ± 1.78			

*x ± SD* mean values and standard deviation

*Mean diff.* mean difference

*Cl* Coinfidence interval

**Fig. 1:**
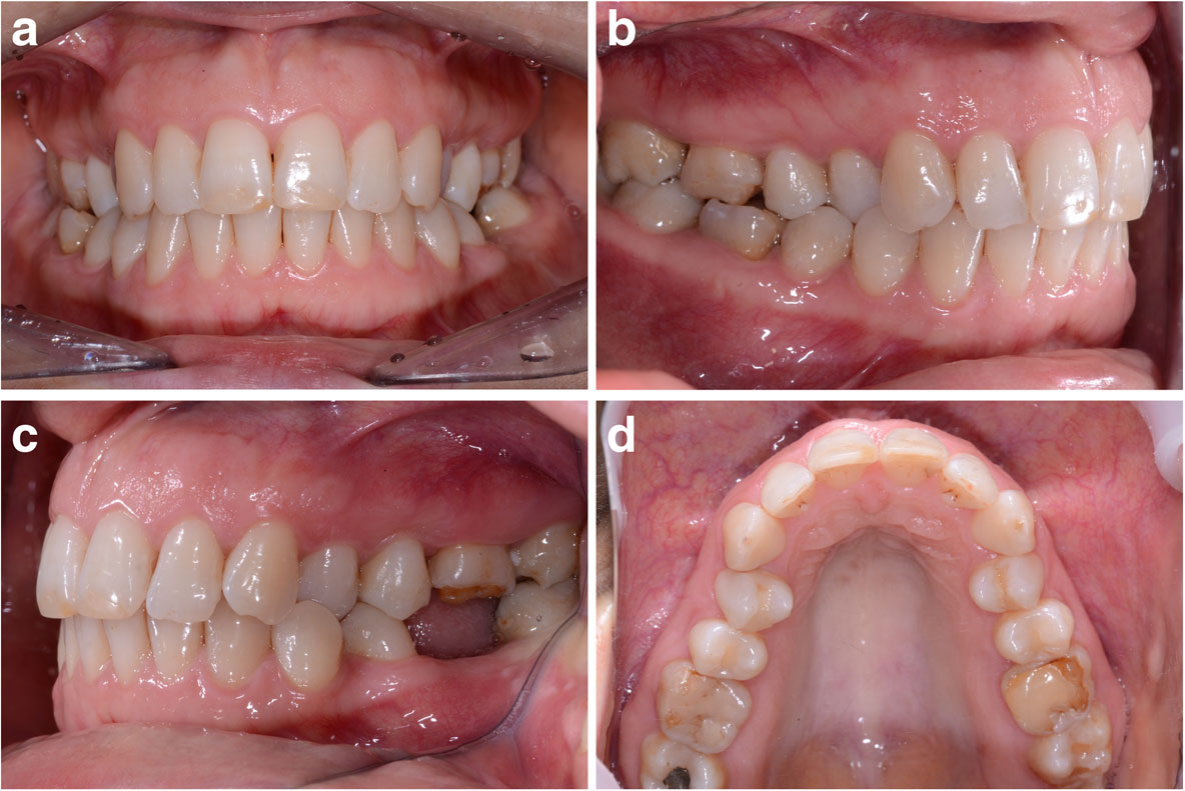
**a-d**. The status before the orthodontic treatment - bilateral crossbite

**Fig. 2:**
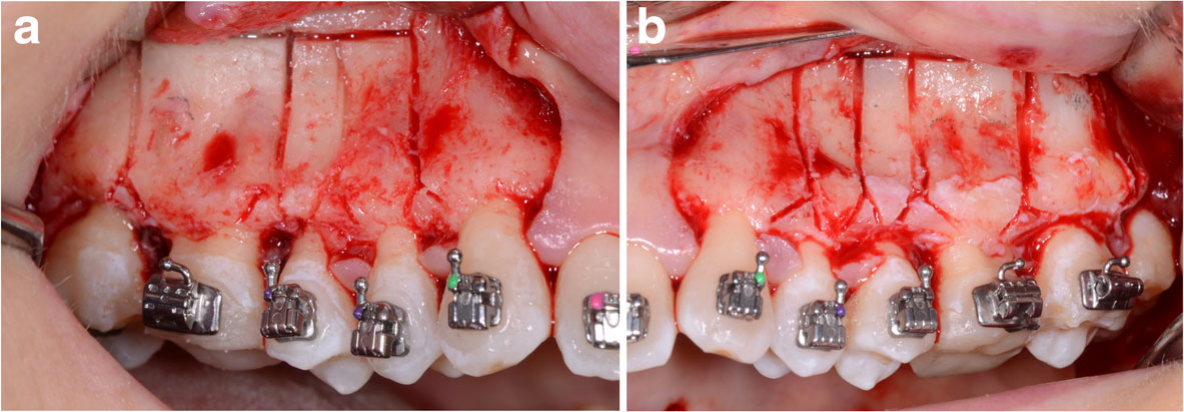
**a-b**. A corticotomy in the area of upper premolars and molars. Incision of the cortical plate in the interdental spaces and above the apexes of the teeth

**Fig. 3:**
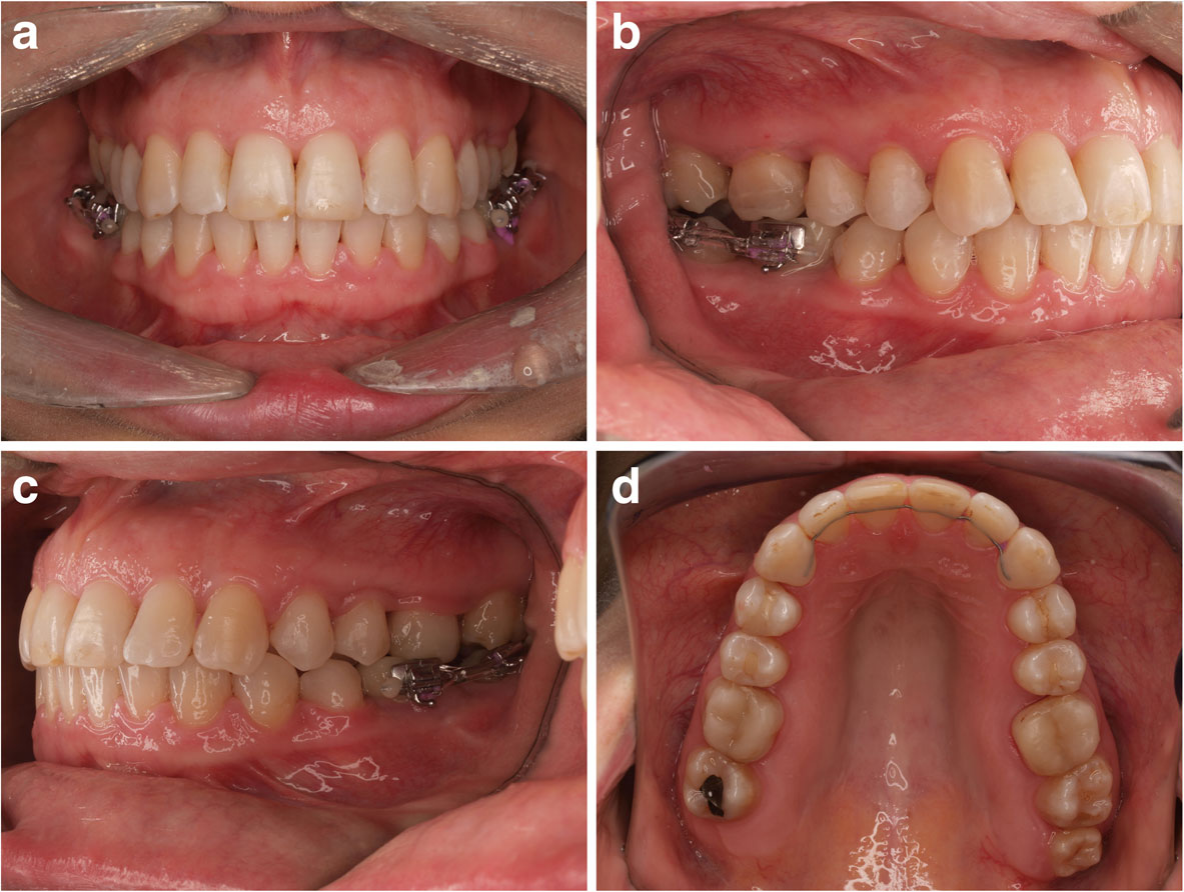
**a-d**. The status imediatelly after orthodontic treatment completion (missing two lower first molars temporarily restored, partial orthodontic appliance left till the moment of the definitive restoration delivery). There are no adverse changes in the position of the gingival margin after labial tooth movement

Miller Class I gingival recessions were found in 12 (7.55%) out of a total 159 assessed teeth. The mean pre-treatment recession height was 0.13 ± 0.47 mm, which decreased to 0.07 ± 0.32 mm after treatment completion, while recession width decreased from 0.21 ± 0.75 mm to 0.10 ± 0.49 mm. No new recessions developed despite the vestibular tooth movement. Out of 12 cases of recession observed before treatment, 5 disappeared, 4 remained unchanged and in 3 GR decreased by 1 mm (Table 3). Figures 1, 2 and 3 show photographic documentation of selected case treatment.

A statistically significant increase was noted in relation to the biotype. Its thickness values rose 3 months post-op (from 1.71 ± 0.52 mm to 1.95 ± 0.54 mm), then decreased 6 months post-op as compared with the 3-month-post-op examinations; then it increased again reaching the maximum mean value (2.03 ± 0.47 mm) in twelfth month, i.e. at the end of the treatment (Table 3). The KT values also increased significantly during the course of the treatment. Its mean value at baseline was 5.02 ± 1.79 mm and rose to 5.12 ± 1.78 mm at the treatment completion (Table 3). No statistically significant differences were observed in GR, RW and PW (Table 3).

## Discussion

The aim of presented study was detailed clinical evaluation of periodontal tissues in adult patients after corticotomy-assisted orthodontic treatment. The results of the study have demonstrated lack of negative influence of the treatment on the periodontal status confirmed by significant reduction in PD and CAL (of 0.26 mm) as well as BS (of 0.27 mm) after treatment. In the available literature Gantes et al. [6], Charavet et al. [24] and Cassetta et al. [25] examined periodontal tissues response to corticotomy-assisted orthodontic treatment. Gantes et al. [6] assessed periodontal parameters - PI, PD, CAL and concluded that corticotomy procedure caused minimal changes in the periodontal attachment apparatus. However, the authors did not provide specific values for periodontal parameters and the study group consisted of only 5 people. Charavet et al. [24] conducted randomized controlled study in the group of 24 adult patients with mild overcrowdings who were randomly allocated to a control group that was treated with conventional orthodontics or a test group that received piezo assisted orthodontics. In both groups, periodontal parameters: PD, PI, papilla bleeding index and recession depth remained unchanged between the baseline and treatment completion time points. Analyze of recession depth in particular cases revealed that recession depth increased only in 3 patients - 2 from the control group and 1 from the test group. This observation is even more interesting because the mean value of the recession depth in the control group was substantially lower than in the test group (2.5 ± 2.3 mm vs 5.7 ± 7.6 mm). In our study, in none of patients new recessions developed despite that orthodontic vestibular movement of teeth was performed. Additionally, 5 of 12 of existing recessions disappeared and 3 reduced of 1 mm. Nevertheless, such changes in the recessions parameters, decrease in the mean values of GR as well as gingival width (respectively 0.06 mm and 0.11 mm) were not statistically significant. Findings of our research may confirm the hypothesis that the potential to increase the post-cortycotomy alveolar volume and cover vital root surfaces can result in repairing pre-existing alveolar dehiscences over the root prominences and lessen a risk of forming new ones, which can contribute to gingival recession [6]. Cassetta et al. [25, 26] assesed modified gingival index (mGI) and probing pocket depth (PPD) before and at the end of the orthodontic treatment assisted with minimally invasive coricotomy performed with the use of printed CAD/CAM surgical guide. The authors didn’t show significant changes in the values of the tested parameters before and after treatment. The mean mGI value at baseline was 0.15 whereas post-op - 0.10. The mean PPD values at baseline and post-op were 1.93 mm and 1.68 mm respectively. The above data showed that both, corticotomy after full-thickness flap elevation and the minimally invasive flapless corticotomy do not adversely affect the status of periodontal tissues. Flapless corticotomy brings also additional benefits - significantly less trauma to the patient and reduced time of the surgery [25, 26].

From the clinical point of view any labial tooth movement should be preceded by a careful examination of the dimensions of the tissue which covers the teeth to be moved. As long as a tooth can be moved within the envelope of the alveolar process, the risk of harmful side-effects on the gingival tissue is minimal, irrespective of the thickness of the soft tissue [27]. If, however, there is danger of alveolar bone dehiscences, thickness of the covering soft tissue must be considered to be a factor leading to gingival recession, both during and after the therapy. Thin gingiva may also serve as a locus minoris resistentiae for gingival recession in the presence of bacterial plaque [28]. Our observations show that the model of corticotomy-facilitated orthodontic treatment may have favourable impact on soft tissue parameters. We have observed a statistically significant increase of KT of 0.1 mm and tissue thickness of 0.32 mm after the treatment. Biotype thickening as well as decrease of the number and dimension of recessions after the treatment suggest that alterations in hard and soft tissues after corticotomy may protect soft tissues position during the teeth movement toward labial direction. Similar findings achieved Liou and Huang [29], who concluded that the periodontal ligament could be rapidly distracted without complications after corticotomy and this technique could be used to generate new bone growth and keratinized gingiva. It seems that this favorable reaction for treatment is due to RAP, which according to Yaffe et al. [12] is essentially a temporary stage of localized soft- and hard tissues remodeling in the process of bringing the surgical site to a normal state and usually takes about four months to heal [30]. It is also possible that the optimistic results obtained are due to the use of piezosurgery device. Dibart el al. [31] has shown that although all corticotomy procedures involve physical injury to the bone, the clinical outcomes may depend on the instrument used. Using an ex vivo calvarial bone organ culture model system, the authors evaluated the biologic response of bone to different corticotomies. Bone injuries were generated in neonatal mice using a piezoelectric knife, a bur, and a handheld screw device. It was demonstrated that the piezoelectric knife led to the most extensive impact in both bone resorption and formation models. Farid et al. [32] arrived at the opposite conclusions whose purpose of the research was to evaluate corticotomy-facilitated orthodontics using piezosurgery versus conventional rotary instruments in mongrel dogs. A statistically significantly higher mean amount of tooth movement (1.6 times faster) for conventional rotary instrument versus the piezosurgery corticotomy technique was observed at all time intervals. Notwithstanding the above controversies, there are other advantages of piezosurgery, i.e. permitting a selective cut of mineralized tissue while preserving soft tissues. Moreover, the major advantages of this technique include high precision, curvilinear design of the osteotomy, less trauma to soft tissues, preservation of neurological and vascular structures, reduced hemorrhage, minimal thermal damage to the bone, as well as overall improvement of healing [33]. However, taking into account the oral health-related quality of life - OHIP-14 (which represents: functional limitation, physical pain, psychological discormfort, physical disability, psychological disability, social disability, and handicap) corticotomy with the use of bur or piezoelectric knife do not differ significantly [34].

As post-corticotomy tooth movement does not have negative effects on the periodontium, our results may also suggest, that there is no need for additional bone augmentation although some authors recommend bone grafting in the area where expansion of the alveolar bone is needed [9, 10, 12]. All the more that Nowzari et al. [13] pointed out that an optimal quantity of bone graft has not been determined yet and more clinical research should be undertaken to do so. Significant confirmation of such suggestion is study done by Chavret et al. [24] in which minimally invasive corticotomy technique - piezocision without hard and soft tissue augmentation was used. No significant increases in dehiscence or fenestration were observed. Additionally, the thickness of the buccal alveolar plate and the bucco-lingual dimensions of the alveolar crest did not significantly change from baseline to the completion of treatment.

The last aspect of our research was the evaluation of interdental papillae. In opposition to other authors who haven’t observed papillae height reduction after osteotomy accelerated orthodontics, we have noticed a statistically significant reduction in papillae height that was also reflected in the bone sounding values [35]. Indeed, mean PH value decreased of 0.82 mm comparing to baseline but it cannot be directly related to corticotomy procedure but rather as the effect of the teeth position changes [36]. Within the course of orthodontic treatment arches were extended, crowded teeth were aligned and unrotated if needed. Alterations of teeth position and arches shape cause papillae remodeling including changes in the distance between papilla tip and interproximal contact point. Considering the fact that the papillae height is influenced by many factors (distance between bone level and approximal contact point, distance between roots at the bone level and divergent roots position), it is not possible to explicitly refer to changes in their height after treatment [36–38]. That is why in our opinion reduction of PH parameter cannot be unequivocally considered as deterioration in interproximal papillae condition.

Summarizing all above data, it should be underlined that the results presented in this article included entire estimation of clinical soft tissues parameters which, according to our knowledge, have not been presented in the literature yet. It was shown up that there was a statistically significant reduction in PD, CAL and BS after the treatment. Statistically significant changes were also noted considering KT and biotype, which increased significantly after utilization of the corticotomy-assisted orthodontic treatment. Therefore, the achieved findings may suggest protective role of corticotomy on soft tissues condition in the course of orthodontic treatment. However, the presented study is burdened with a limitation resulting from the lack of a control group in which patients were treated orthodontically without additional corticotomy. In future studies it would be also helpful to analyze CBCT images to assess the changes in the bone morphology following the piesosurgery-assisted orthodontics.

## Conclusion

The corticotomy–assisted orthodontic treatment does not jeopardize a periodontal clinical status. Since the currently available literature misses detailed studies on the periodontal changes which occur after the procedure, there is a need to continue studies on a larger number of cases with a control group and a longer follow-up time to find out about the post-treatment periodontal tissue changes and stability.

## Abbreviations

BS: Bone sounding
CAL: Clinical attachment level
CEJ: Cemento-enamel junction
FMBOP: Full mouth bleading on probing
FMPI: Full mouth plaque index
GR: Gingival recession height
KT: Keratinized tissue
MTDLD: Monocortical tooth dislocation and ligament distraction
PAOO: Periodontally accelerated osteogenic orthodontics
PD: Probing depth
PH: Papilla height
PW: Papilla width
RAP: Regional accelerated phenomenon
RW: Recession width

## References

[1] Patel N (2014). Corticotomy assisted orthodontic: a review of surgical technique and literature. OA Dentistry.

[2] Hassan AH, Al-Fraidi AA, Al-Saeed SH (2010). Corticotomy-assisted orthodontic treatment: review. Open Dent J.

[3] Oliveira DD, Oliveira BF, Soares RV (2010). Alveolar corticotomies in orthodontics: indications and effects on tooth movement. Dental Press J Orthod.

[4] Buschang PH, Phillip M, Campbell PM, Ruso S (2012). Accelerating tooth movement with Corticotomies: is it possible and desirable?. Semin Orthod.

[5] Köle H (1959). Surgical operations on the alveolar ridge to correct occlusal abnormalities. Oral Surg Oral Med Oral Pathol.

[6] Gantes B, Rathbun E, Anholm M (1990). Effects on the periodontium following corticotomy-facilitated orthodontics. Case reports. J Periodontol.

[7] Wilcko WM, Wilcko TM, Bouquot JE, Ferguson DJ (2001). Rapid orthodontics with alveolar reshaping: two case reports of decrowding. Int J Periodontics Restorative Dent.

[8] Wilcko MT, Wilcko WM, Bissada NF (2008). An evidence based analysis of periodontally accelerated orthodontic and osteogenic techniques: a synthesis of scientific perspectives. Semin Orthod.

[9] Wilcko MT, Wilcko WM, Pulver JJ, Bissada NF, Bouquot JE (2009). Accelerated osteogenic orthodontics technique: a 1-stage surggically facilitated rapid orthodontic technique with alveolar augmentation. J Oral Maxillofac Surg.

[10] Murphy KG, Wilcko MT, Wilcko WM, Ferguson DJ (2009). Periodontal accelerated Osteogenic orthodontics: a description of the surgical technique. J Oral Maxillofac Surg.

[11] Frost HM (1983). The regional acceleratoty phenomenon: a review. Henry Ford Hosp Med J.

[12] Yaffe A, Fine N, Binderman I (1994). Regional accelerated phenomenon in the mandible following mucoperiosteal flap surgery. J Periodontol.

[13] Nowzari H, Yorita FK, Chang HC (2008). Periodontally accelerated osteogenic orthodontics combined with autogenous bone grafting. Compend Contin Educ Dent.

[14] Sebaoun JD, Kantarci A, Turner JW, Carvalho RS, Van Dyke TE, Ferguson DJ (2008). Modeling of trabecular bone and lamina dura following selective alveolar decortication in rats. J Periodontol.

[15] Lee W, Karapetyan G, Moats R, Yamashita DD, Moon HB, Ferguson DJ, Yen S (2008). Corticotomy−/osteotomy-assisted tooth movement microCTs differ. J Dent Res.

[16] Vercellotti T, Podesta A (2007). Orthodontic microsurgery: a new surgically guided technique for dental movement. Int J Periodontics Restorative Dent.

[17] Dibart S, Sebaoun JD, Surmenian J (2009). Piezocision: a minimally invasive, periodontally accelerated orthodontic tooth movement procedure. Compend Contin Educ Dent.

[18] Nazarov AD, Ferguson DJ, Wilcko WM, Wilcko MT (2004). Improved orthodontic retention following corticotomy using ABO objective grading system. J Dent Res.

[19] Fischer TJ (2007). Orthodontic treatment acceleration with corticotomy assisted exposure of palatally impacted canines. Angle Orthod.

[20] Mostafa YA, Mohamed Salah Fayed M, Mehanni S, ElBokle NN, Heider AM (2009). Comparison of corticotomy-facilitated vs standard tooth-movement techniques in dogs with miniscrews as anchor units. Am J Orthod Dentofac Orthop.

[21] Dorfman HS, Turvey TA (1979). Alterations in osseous crestal height following interdental osteotomies. Oral Surg Oral Med Oral Pathol.

[22] Kwon HJ, Pihlstrom B, Waite DE (1985). Effects on the periodontium of vertical bone cutting for segmental osteotomy. J Oral Maxillofac Surg.

[23] Cortellini P, Pini Prato G, Tonetti MS (1995). The modified papilla preservation technique. A new surgical approach for interproximal regenerative procedures. J Periodontol.

[24] Charavet C, Lecloux G, Bruwier A, Rompen E, Maes N, Limme N, Lambert F (2016). Localized piezoelectric alveolar decortication for orthodontic treatment in adults: A randomized controlled trial. J Dent Res.

[25] Cassetta M, Giansanti M, Di Mambro A, Calasso S, Barbato E (2016). Minimally invasive corticotomy in orthodontics using a three-dimensional printed CAD/CAM surgical guide. Int J Oral Maxillofac Surg.

[26] Cassetta M, Pandolfi S, Giansanti M (2015). Minimally invasive corticotomy in orthodontics: a new technique using a CAD/CAM surgical template. Int J Oral Maxillofac Surg.

[27] Borzabadi-Farahani A (2012). A review of the oral health-related evidence that supports the orthodontic treatment need indices. Prog Orthod.

[28] Wennström JL, Lindhe J, Sinclair F (1987). Some periodontal tissue reactions to orthodontic tooth movement in monkeys. J Clin Periodontol.

[29] Liou EJ, Huang CS (1998). Rapid canine retraction through distraction of the periodontal ligament. Am J Orthod Dentofac Orthop.

[30] Abbas IT, Moutamed GM (2012). Acceleration of orthodontic tooth movement by alveolar corticotomy using piezosurgery. J Am Sci.

[31] Dibart S, Alasmari A, Zanni O, Salih E (2016). Effect of Corticotomies with different instruments on cranial bone biology using an ex vivo Calvarial bone organ culture model system. Int J Periodontics Restorative Dent.

[32] Farid KA, Mostafa YA, Kaddah MA, El-Sharaby FA (2014). Corticotomy-facilitated orthodontics using piezosurgery versus rotary instruments: an experimental study. J Int Acad Periodontol.

[33] Hennet P (2015). Piezoelectric bone surgery: a review of the literature and potential applications in veterinary oromaxillofacial surgery. Front Vet Sci.

[34] Cassetta M, Di Carlo S, Giansanti M, Pompa V, Pompa G, Barbato E (2012). The impact of osteotomy technique for corticotomy-assisted orthodontic treatment (CAOT) on oral health-related quality of life. Eur Rev Med Pharmacol Sci.

[35] Bertossi D, Vercellotti T, Podesta A, Nocini PF (2011). Orthodontic microsurgery for rapid dental repositioning in dental malpositions. J Oral Maxillofac Surg.

[36] Cho HS, Jang HS, Kim DK, Park JC, Kim HJ, Choi SH, Kim CK, Kim BO (2006). The effects of interproximal distance between roots on the existence of interdental papillae according to the distance from the contact point to the alveolar crest. J Periodontol.

[37] Sharma AA, Park JH (2010). Esthetic considerations in interdental papilla: remediation and regeneration. J Esthet Restor Dent.

[38] Martegani P, Silvestri M, Mascarello F, Scipioni T, Ghezzi C, Rota C, Cattaneo V, Kim BO (2007). Morphometric study of the interproximal unit in the esthetic region to correlate anatomic variables affecting the aspect of soft tissue embrasure space. J Periodontol.

